# DNA methylation regulates expression of *VEGF-R2 *(*KDR*) and *VEGF-R3 *(*FLT4*)

**DOI:** 10.1186/1471-2407-12-19

**Published:** 2012-01-17

**Authors:** Hilmar Quentmeier, Sonja Eberth, Julia Romani, Herbert A Weich, Margarete Zaborski, Hans G Drexler

**Affiliations:** 1 Department of Human and Animal Cell Cultures, German Collection of Microorganisms and Cell Cultures, Braunschweig, Germany; 2 Department of Hematology and Oncology, Georg-August-University Göttingen, University Medical Center, Göttingen, Germany; 3 Department of Gene Regulation and Differentiation, Helmholtz Centre for Infection Research, Braunschweig, Germany

## Abstract

**Background:**

Vascular Endothelial Growth Factors (VEGFs) and their receptors (VEGF-Rs) are important regulators for angiogenesis and lymphangiogenesis. VEGFs and VEGF-Rs are not only expressed on endothelial cells but also on various subtypes of solid tumors and leukemias contributing to the growth of the malignant cells. This study was performed to examine whether *VEGF-R2 *(*KDR*) and *VEGF-R3 *(*FLT4*) are regulated by DNA methylation.

**Methods:**

Real-time (RT) PCR analysis was performed to quantify *KDR *and *FLT4 *expression in some ninety leukemia/lymphoma cell lines, human umbilical vein endothelial cells (HUVECs) and dermal microvascular endothelial cells (HDMECs). Western blot analyses and flow cytometric analyses confirmed results at the protein level. After bisulfite conversion of DNA we determined the methylation status of *KDR *and *FLT4 *by DNA sequencing and by methylation specific PCR (MSP). Western blot analyses were performed to examine the effect of VEGF-C on p42/44 MAPK activation.

**Results:**

Expression of *KDR *and *FLT4 *was observed in cell lines from various leukemic entities, but not in lymphoma cell lines: 16% (10/62) of the leukemia cell lines expressed *KDR*, 42% (27/65) were *FLT4 *positive. None of thirty cell lines representing six lymphoma subtypes showed more than marginal expression of *KDR *or *FLT4*. Western blot analyses confirmed KDR and FLT4 protein expression in HDMECs, HUVECs and in cell lines with high VEGF-R mRNA levels. Mature VEGF-C induced p42/44 MAPK activation in the KDR^- ^/FLT4^+ ^cell line OCI-AML1 verifying the model character of this cell line for VEGF-C signal transduction studies. Bisulfite sequencing and MSP revealed that GpG islands in the promoter regions of *KDR *and *FLT4 *were unmethylated in HUVECs, HDMECs and *KDR^+ ^*and *FLT4^+ ^*cell lines, whereas methylated cell lines did not express these genes. In hypermethylated cell lines, *KDR *and *FLT4 *were re-inducible by treatment with the DNA demethylating agent 5-Aza-2'deoxycytidine, confirming epigenetic regulation of both genes.

**Conclusions:**

Our data show that VEGF-Rs *KDR *and *FLT4 *are silenced by DNA methylation. However, if the promoters are unmethylated, other factors (e.g. transactivation factors) determine the extent of *KDR *and *FLT4 *expression.

## Background

Vascular endothelial growth factors (VEGFs) and their corresponding receptors (VEGF-Rs) are important regulators of angiogenesis and lymphangiogensis. VEGF-A binds VEGF-R1 (FLT1) and VEGF-R2 (KDR). Both tyrosine kinase receptors are expressed on blood vessel endothelial cells. VEGF-C and VEGF-D bind to VEGF-R3 (FLT4) and the fully processed, mature forms also to KDR. FLT4 is primarily expressed on cells of the lymphatic endothelium [1]. VEGFs and VEGF-Rs are important for vessel formation in healthy individuals, but also for tumor angiogenesis [2]. Moreover, the VEGF-Rs are not only expressed on endothelia, but also on different types of solid tumor cells and on leukemic cells [3-11]. The interaction of receptors with their ligands mediates survival and can lead to proliferation of the malignant cells [2,12].

Even twenty years after their discovery, little is known about the regulation of the three VEGF-Rs. On the transcriptional level, NF-κB and the NF-κB target *Prox1 *have been described as activators of *FLT4 *in lymphatic endothelial cells [13]. Epigenetic mechanisms contribute to the regulation of *FLT1 *and *KDR *but this is not investigated in great detail [14,15].

We set out to test whether DNA methylation is also responsible for the silencing of *FLT4*. We determined the methylation status of *KDR *and *FLT4 *in human umbilical vein endothelial cells (HUVECs), dermal microvascular endothelial cells (HDMECs) and in a large panel of leukemia and lymphoma cell lines. Confirming that expression of *KDR *and *FLT4 *is epigenetically regulated, we observed an inverse correlation between promoter methylation and receptor expression. Furthermore, the demethylating agent 5-Aza-2'deoxycytidine (5-Aza-dC) induced expression of *KDR *and *FLT4 *in methylated, but not in unmethylated cell lines.

## Methods

### Cell lines and primary cell cultures

The cell lines in this study were taken from the stock of the cell bank (DSMZ--German Collection of Microorganisms and Cell Cultures; http://www.dsmz.de). Detailed references and cultivation protocols have been described previously [16]. Primary HDMECs were purchased from Clonetics/Lonza (Verviers, Belgium). Primary HUVECS (pooled) were purchased from PromoCell (Heidelberg, Germany). HDMECs and HUVECs were cultured in endothelial cell growth medium MV (Promo Cell).

### CpG island search

CpG island search was done with Methyl Primer Express v1.0 software and EMBOSS CpG plot (http://www.ebi.ac.uk/Tools/emboss/cpgplot/index.html). The criteria for an island were: GC content > 50%; CpG observed *versus *CpG expected ratio > 0.6, length > 100 bp.

### Methylation-specific polymerase chain reaction (MSP)

Bisulfite conversion of DNA was performed as described by the supplier (EpitTect Bisulfite Kit, Qiagen, Hilden, Germany). For detecting *FLT4 *and *KDR *promoter methylation, we performed nested PCR with first round primers (*FLT4 *BSP fwd 5'-AAA TAT TTG GGG GAG TTT TAA A-3', *FLT4 *BSP rev 5'-CCC AAT CTC AAA AAT AAA CAA A-3'; *KDR *BSP fwd 5'-AAG TTG TTG TTT TGG GAT GTT T-3', *KDR *BSP rev 5'-AAA TAA ACT CCT TAC CCA CAA A-3') amplifying converted DNA independently of the methylation status (bisulfite-specific PCR or BSP; annealing temp.: 54.9°C for *FLT4 *BSP, 54.7°C for *KDR *BSP, 35 cycles), and second round primers for M- and U-PCR specifically recognizing the methylated or unmethylated versions of the promoter (*FLT4 *M fwd 5'-GTC GGT TAT TTC GGG TGT TTC -3', *FLT4 *M rev 5'-AAT ATC GAC GAA CAA TAT CGA CG-3', *FLT4 *U fwd 5'-GGG TTG GTT ATT TTG GGT GTT TT-3', *FLT4 *U rev 5'-ACA CAA TAT CAA CAA ACA ATA TCA ACA-3', *KDR *M fwd 5'-CGT TTT CGC GTT TTA GAG TTT C-3', *KDR *M rev 5'-GCG CAA ATA ATA CCC GAC G-3', *KDR *U fwd 5'-TTT TGT TTT TGT GTT TTA GAG TTT T-3', *KDR *U rev 5'-ACA CAC AAA TAA TAC CCA ACA-3'). PCR products of the initial BSP were diluted 1:100 to 1:4.000 for subsequent M- and U-PCR. Annealing temperature was 61.2°C for *FLT4 *M- and U-PCR, 58°C for *KDR *M- and U-PCR with 30 cycles each. Epitect PCR Control DNA (Qiagen) was used as control for methylated and unmethylated templates.

### Bisulfite sequencing

To confirm the methylation status of the *FLT4 *and *KDR *promoters, genomic DNA was bisulfite converted according to the manufacturer's instructions (Qiagen). Subsequently, amplification of the promoter regions (*FLT4*: 337 bp; *KDR*: 612 bp) was performed using BSP primers, specifically binding bisulfite converted DNA (for primer sequence and PCR conditions see MSP section). Resulting PCR products were purified, cloned into the pGEM-TEasy vector (Promega, Madison, WI, USA) and sequenced. Sequences were evaluated using BiQ Analyzer (http://biq-analyzer.bioinf.mpi-sb.mpg.de) and had to conform to at least 90% bisulfite conversion rate. In addition, identical clones were excluded from the analysis.

### Gene expression analyses

Quantitative PCR was performed on a 7500 Applied Biosystems (Darmstadt, Germany) real-time PCR system using the manufacturer's protocol. RNA was prepared using the RNeasy Mini kit (Qiagen). This kit includes a DNase digestion step to avoid false positives resulting from contaminating genomic DNA. For mRNA quantification, reverse transcription was performed using the SuperScript II reverse transcriptase kit (Invitrogen, Karlsruhe, Germany). TaqMan probes (Applied Biosystems) were used to quantify human *FLT4 *(Hs 01047677 m1) and *KDR *(Hs 00911700 m1) expression levels with *TATA box binding protein *(*TBP*) as endogenous control. For *interferon gamma inducible protein 10 *(*IP-10*) and *tumor necrosis factor alpha *(*TNFα*), SYTO-82 (Molecular Probes, Leiden, Netherlands) was used as fluorescent dye, ImmoMix (Biline, Luckenwalde, Germany) as PCR master mix, and *ribosomal protein S9 *(*RPS9*) as endogenous control. The following primers were used: *TNFα *exon 2 fwd 5'-CCC CAG GGA CCT CTC TCT AA-3', *TNFα *exon 3 rev 5'-TGG GCT ACA GGC TTG TCA CT-3'; *IP-10 *exon 1 fwd 5'-GCC ATT CTG ATT TGC TGC CTT A -3', *IP-10 *exon 2 rev 5'-TGA TGC AGG TAC AGC GTA CAG-3'; *RPS9 *exon 2 fwd 5'-GGG AAG CGG AGC CAA CAT G-3', *RPS9 *exon 3 rev 5'-GTT TGT TCC GGA GCC CAT ACT-3'. Relative expression levels were calculated using the ΔΔCt-method.

### [^3^H]-Thymidine uptake

Assays of [^3^H]-thymidine incorporation were executed as follows: 1.25 × 10^4 ^cells (in 100 μl) were seeded in triplicate in 96-well flat-bottom microtiter cell culture plates. Inhibitors were added as 2x concentrated solution in a 100 μl volume. For the last 3 h of the incubation period, 1 μCi [^3^H]-thymidine (Hartmann Analytic, Braunschweig, Germany) was added to each well.

### Western blot analysis, antibodies, reagents

Samples were prepared as described previously [17]. Anti FLT4, ERK and pERK antibodies were purchased from Santa Cruz (Heidelberg, Germany). Anti IκB, pIκB, KDR, p38 MAPK and pp38 MAPK antisera were obtained from Cell Signalling (New England Biolabs, Frankfurt, Germany). The anti GAPDH monoclonal antibody (mAb) was purchased from Abcam (Cambridge, UK). Specific bands on nitrocellulose membranes were visualized with the biotin/streptavidin-horseradish peroxidase system (Amersham, Freiburg, Germany) in combination with the "Renaissance Western Blot Chemoluminescence Reagent" protocol (Perkin Elmer, Waltham, MA, USA). Synthetic macrophage activating factor of 2 kDa molecular mass (MALP-2) was a gift from P. Mühlradt to H. Weich. The preparation was free of endotoxin.

### Analysis of FLT4 and KDR protein expression by flow cytometry

For detection of CD31 (Becton Dickinson Biosciences, Heidelberg, Germany), FLT4 (R&D Systems, Wiesbaden, Germany), KDR (Reliatech, Wolfenbüttel, Germany) and podoplanin (Reliatech) on the cell surface, cells were washed and incubated with the mouse mAb or with the isotope-matched control mouse immunoglobulin (BD Biosciences) for 30 min at 4°C. Subsequently, cells were treated with FITC conjugated anti-mouse secondary Ab (Biozol, Eching, Germany) and propidium iodide. Labeled cells were analyzed on a FACSCalibur (BD Biosciences) using CellQuest Pro software.

### Treatment with DNA demethylating agent 5-Aza-2'-deoxycytidine (5-Aza-dC)

5-Aza-dC (Sigma Aldrich, Taufkirchen, Germany) dissolved in DMSO was used to verify the effect of methylation on expression of *FLT4 *and *KDR*. Cells were seeded at a cell density of 5 × 10^5 ^cells/ml, 5-Aza-dC was added at a final concentration of 5 μM. Control cells were treated with 0.05% DMSO. After 2 d, half of the medium was replenished with medium with/without 5-Aza-dC (5 μM). After 3 d, cells were harvested to prepare RNA and protein.

## Results and Discussion

### Expression of *KDR *and *FLT4 *in leukemia and lymphoma cell lines

The VEGF-Rs *KDR *(*VEGF-R2*) and *FLT4 *(*VEGF-R3*) are not only expressed on blood endothelial and lymphendothelial cells, but also on solid tumors and leukemias. Leukemia-derived VEGFs may induce the growth of leukemic cells in an autocrine or paracrine fashion [7,10,18,19]. The promoters of VEGF-Rs and their ligands contain CpG islands, regulatory regions that are typically methylated in epigenetically silenced genes [14]. Recent reports show that expression of *FLT1 *and *KDR *are controlled by promoter methylation [14,15]. However, only a limited number of leukemia and lymphoma cell lines have been tested for *VEGF-R *expression and promoter methylation hitherto.

**Figure 1:**
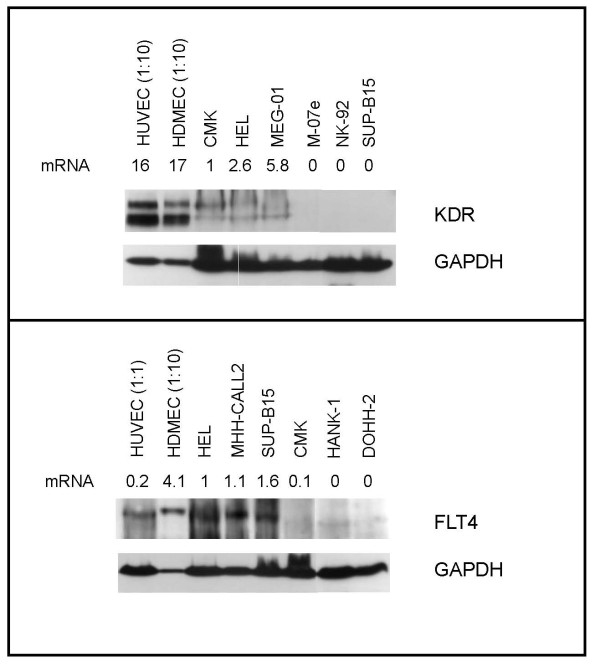
**KDR and FLT4 in HDMECs, HUVECs and leukemia cell lines**. *KDR *and *FLT4 *mRNA expression levels were determined by quantitative real-time PCR and indicated underneath the cell line name. *TBP *expression was used as endogenous control and cell lines CMK (KDR) and HEL (FLT4) were used for normalization. KDR protein expression levels--determined by Western blot analysis--are higher in HDMECs and HUVECs than in positive cell lines (note 1:10 lysate dilution in primary cells). FLT4 protein expression is higher in HDMECs than in HUVECs (note 1:10 lysate dilution in HDMECs) and in positive cell lines. Protein expression corresponds to mRNA expression pattern.

To find model systems for *VEGF-R *regulation, we tested some ninety leukemia and lymphoma cell lines for *KDR *and *FLT4 *mRNA expression. Both genes were regularly expressed in leukemia but not in lymphoma cell lines: 10/62 (16%) cell lines from various leukemic entities expressed *KDR*, 27/65 (42%) expressed *FLT4 *(Table 1). In contrast, 0/30 lymphoma cell lines expressed *KDR*, and only 1/30 (3%) expressed *FLT4 *(Table 1). Cell lines with high *VEGF-R *transcript levels expressed also the corresponding proteins: cell lines CMK, HEL and MEG-01 expressed KDR, whereas cell lines HEL, MHH-CALL2, OCI-AML1 and SUP-B15 were FLT4 positive (Figure 1, Table 2).

**Table 1:** *VEGF-R *mRNA expression in leukemia and lymphoma cell lines

			KDR			
	+++	++	+	(+)	-	∑
AML	0	2	0	3	16	21
pre-B ALL	0	0	0	1	10	11
T-ALL	0	0	0	2	9	11
NK	0	0	0	0	5	5
CML	1	0	0	1	12	14
HL	0	0	0	0	5	5
ALCL	0	0	0	0	5	5
BL	0	0	0	0	5	5
DLBCL	0	0	0	0	5	5
FL	0	0	0	0	5	5
MCL	0	0	0	0	5	5
∑	1	2	0	7	82	92

			FLT4			
	+++	++	+	(+)	-	∑

						
AML	0	2	4	3	14	23
pre-B ALL	0	3	1	3	4	11
T-ALL	0	0	2	4	5	11
NK	0	0	0	0	5	5
CML	0	1	1	3	10	15
HL	0	0	0	1	4	5
ALCL	0	0	0	0	5	5
BL	0	0	0	0	5	5
DLBCL	0	0	0	0	5	5
FL	0	0	0	0	5	5
MCL	0	0	0	0	5	5
∑	0	6	8	14	67	95

*KDR *and *FLT4 *mRNA expression levels were determined by quantitative real-time PCR. *TBP *expression was used as endogenous control and cell lines CMK (*KDR*) and HEL (*FLT4*) were used for normalization. Relative quantification: +++ ≥ 5; ++ ≥ 1; + ≥ 0.2; (+) ≥ 0.04;- < 0.04. AML, acute myeloid leukemia; ALL, acute lymphoblastic leukemia; NK, natural killer leukemia; CML, chronic myeloid leukemia; HL, Hodgkin lymphoma; ALCL, anaplastic large cell lymphoma; BL, Burkitt lymphoma; DLBCL, diffuse large B cell lymphoma; FL, follicular lymphoma, MCL, mantle cell lymphoma

**Table 2:** Promoter methylation status and expression levels of *KDR *and *FLT4*

		*KDR*			*FLT4*	
	MSP	mRNA	protein	MSP	mRNA	protein
697	M	0.08	neg	M/U	0	neg
ALL-SIL	M	0	neg	U	0.3	neg
AP-1060	M/U	0	n.d.	M/U	0	n.d.
BV-173	M	0	neg	U	1.4	pos
CMK	M/U	1	pos	M	0.1	neg
DOHH-2	M/U	0	neg	M/U	0	neg
EM-2	M/U	0	neg	M	0	neg
HANK-1	M	0	neg	M	0	neg
HEL	U	2.6	pos	U	1	pos
HL-60	M	0	neg	M	0.1	neg
JURL-MK1	U	0.15	n.d.	n.d.	n.d.	n.d.
L-82	M	0	n.d.	M	0	n.d.
LOUCY	n.d.	0.13	neg	M/U	0	neg
M-07e	M/U	0.03	neg	U	0.1	neg
MEG-01	U	5.8	pos	U	0.2	pos
MEGAL	M	0	neg	M/U	0.3	neg
MHH-CALL2	M/U	0	neg	U	1.1	pos
MHH-TALL1	M	0.08	n.d.	n.d.	n.d.	n.d.
MOLT-4	M	0	n.d.	M/U	0	n.d.
MUTZ-3	n.d.	n.d.	n.d.	U	0.6	neg
MUTZ-8	n.d.	n.d.	n.d.	M/U	0.3	neg
NK-92	M	0	neg	M/U	0	neg
OCI-AML1	M/U	0	neg	M/U	1.3	pos
SC-1	M	0	n.d.	M	0	n.d.
SKNO-1	U	0.08	neg	n.d.	n.d.	n.d.
SUP-B15	M	0	neg	U	1.6	pos
TF-1	U	0.1	pos	U	0	neg
THP-1	M/U	0	n.d.	M/U	0	n.d.

*KDR *and *FLT4 *methylation was determined by MSP. M, positive in M-PCR; U, positive in U-PCR, M/U positive in M- and in U-PCR; n.d., not done. *VEGF-R *mRNA expression levels were determined by quantitative real-time PCR. Cell lines CMK (*KDR*) and HEL (*FLT4*) were used for normalization (set to 1). Protein expression was done by Western blot analysis. The accuracy of *KDR *M-PCR (mRNA positivity > 0.1) is 88%, the accuracy of *FLT4 *M-PCR (mRNA positivity > 0.1) is 80%

### OCI-AML1: a model system for VEGF-C induced cell signaling

**Figure 2:**
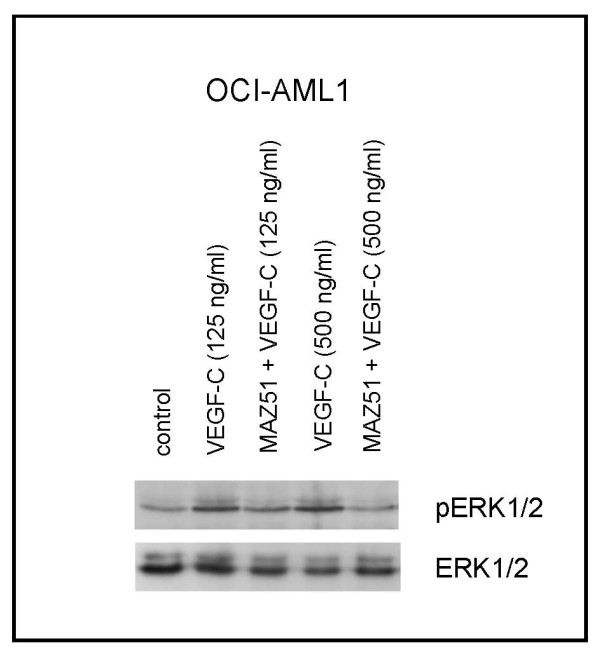
**Phosphorylation level of ERK1/2 in VEGF-C responsive OCI-AML1 cells**. The cytokine-responsive cell line OCI-AML1 was cytokine-starved for 18 h, then stimulated for 5 min with VEGF-C. Pretreatment with the FLT4 inhibitor MAZ51 (20 μM, 1 h) prevented VEGF-C induced ERK1/2 phosphorylation as shown by Western blot analysis.

Cytokine-dependent cell lines have often and successfully been used as model systems for signal transduction studies. In contrast to primary cells, no contaminating cell fraction effects "false" signals in cell lines, and in contrast to cytokine-independently growing cell lines, cytokine starvation silences the relevant enzymes in cytokine-dependent cell lines. We chose cell line OCI-AML1 as this was the only cytokine dependent, *FLT4 *positive cell line tested (Table 2). The cytokine response profile of this cell line has been published previously [20]. Cell line OCI-AML1 did not show a proliferative response on VEGF-C (data not shown). However, short-term (5 min) stimulation with VEGF-C induced phosphorylation of ERK1/2 (Figure 2). Preincubation with the FLT4 inhibitor MAZ51 inhibited this effect, confirming the specificity of the VEGF-C induced EKR1/2 activation (Figure 2). ERK1/2 phosphorylation was tested because the p42/44 MAPK pathway is a known FLT4 target [21,22]. The results of cell signaling experiments shown in Figure 2 confirm that cell line OCI-AML1 is a model system for FLT4 signaling, expecially as KDR, the second receptor for VEGF-C is not expressed in this cell line (Table 2).

### KDR: promoter methylation and gene expression

**Figure 3:**
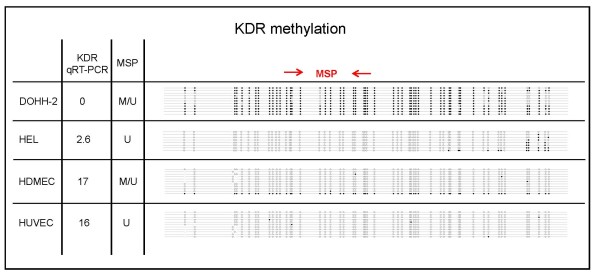
**Bisulfite sequencing of the *KDR *promoter**. A CpG island is located between -1231 and 1125 relative to the ATG codon of *KDR*. The 3' part of the *KDR *promoter region and exon 1 (612 bp, 53 CpG sites) were sequenced after bisulfite conversion of DNA from cell lines DOHH-2 (*KDR *negative) and HEL (*KDR *positive) as well as from HDMECs and HUVECs. Each line depicts a sequenced clone representing the methylation status of an individual allele. CpGs are represented as open dots (if unmethylated) or filled dots (if methylated). Results of qRT-PCR and methylation specific PCR (MSP) are shown on the left hand side. M: signal in M-PCR; U: signal in U-PCR.

**Figure 4:**
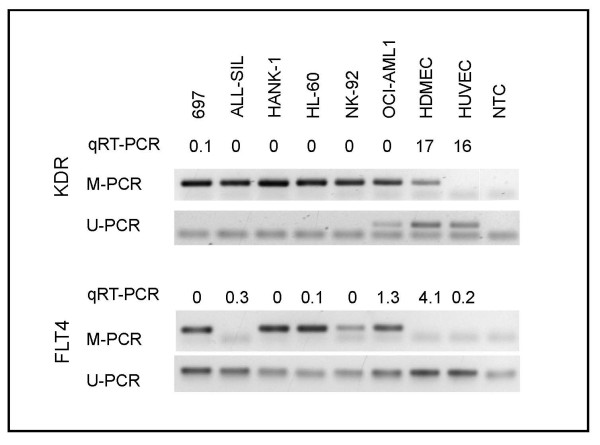
**Methylation status of *KDR *and *FLT4 *in cell lines and primary cells**. The methylation status of *KDR *and *FLT4 *in leukemia cell lines and in HDMECs and HUVECs was analyzed by MSP after bisulfite conversion of the DNA. Agarose gels of *KDR *and *FLT4 *M- and U-PCR are shown. A complete list of results is shown in Table 2. NTC, non template control.

To test whether *KDR *is epigenetically regulated, we performed bisulfite sequencing of *KDR *negative and positive cell lines and of primary endothelial cells. The *KDR *negative cell line DOHH-2 had a highly methylated *KDR *promoter, the *KDR *positive cell line HEL was nearly unmethylated (Figure 3). Largely unmethylated were also HDMECs and HUVECs, both expressing *KDR *(Figure 3). To assess the *KDR *methylation status for a larger number of cell lines, we performed methylation-specific PCR (MSP), a technique less costly and laborious than bisulfite sequencing. The majority of *KDR *negative cell lines were methylated, *KDR *positive HUVECs were unmethylated (Figure 4). However, even HDMECs were U-and M-PCR positive although they expressed high *KDR *levels and although only a small minority of clones were methylated according to sequencing analysis (Figures 3 and 4). Apparently, a low proportion of methylated CpGs was sufficient to yield signals in the M-PCR. The same was true for U-PCR: the *KDR *negative cell line DOHH-2 - highly methylated according to the results of bisulfite sequencing--showed signals in M- and in U-PCR (Figure 3, Table 2).

In spite of the high sensitivity--a certain drawback of the PCR-based MSP technique--the accuracy of *KDR *M-PCR was 88% supporting the notion that *KDR *expression is regulated by DNA methylation (Table 2).

### FLT4: promoter methylation and gene expression

**Figure 5:**
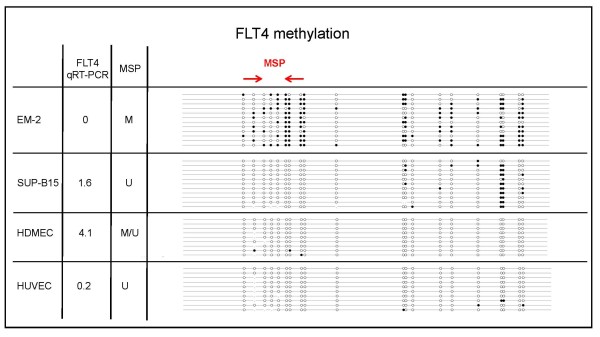
**Bisulfite sequencing of the *FLT4 *promoter**. A CpG island is located between -1231 and 769 relative to the ATG codon of *FLT4*. Part of the promoter region (337 bp, 20 CpG sites) was sequenced from cell lines EM-2 (*FLT4 *negative) and SUP-B15 (*FLT4 *positive) as well as from HDEMCs and HUVECs. Each line depicts a sequenced clone representing the methylation status of an individual allele. CpGs are represented as open dots (if unmethylated) or filled dots (if methylated). Results of qRT-PCR and methylation specific PCR (MSP) are shown on the left hand side. M: signal in M-PCR; U: signal in U-PCR.

Bisulfite sequencing and BSP analysis were also performed to analyze the methylation status of *FLT4 *in cell lines, HUVECs and HDMECs. Results of bisulfite sequencing showed that *FLT4 *was largely methylated in the *FLT4 *negative cell line EM-2 and unmethylated in the *FLT4 *positive cell line SUP-B15 as it was in HUVECs and HDMECs (Figure 5). MSP analysis confirmed that *FLT4 *exhibited the inverse correlation between promoter methylation and gene expression that is indicative for epigenetic regulation (Figure 4, Table 2). However, the accuracy of *FLT4 *M-PCR (80%) was lower than for *KDR *M-PCR (88%). Of note was also that TF-1 cells did not express *FLT4 *although the promoter was unmethylated (Table 2). These data suggested that regulatory mechanisms other than DNA methylation are also important for the regulation of *FLT4*.

### Effect of DNA demethylating agent 5-Aza-dC on expression of KDR and FLT4

To test whether *KDR *and *FLT4 *were silenced by promoter methylation, we treated methylated and unmethylated cell lines with the DNA demethylating agent 5-Aza-dC. In 4/5 *KDR*-negative cell lines, expression of *KDR *was induced by DNA demethylation (Table 3). *FLT4 *expression was upregulated in 4/4 negative cell lines (Table 3). *KDR *and *FLT4 *expression in positive cell lines were not affected (Table 3). Although these results confirmed that promoter methylation plays a role for the regulation of these *VEGF-Rs*, we also noted substantial differences in the levels of 5-Aza-dC-triggered gene induction between different cell lines (Table 3). Furthermore, even in the most sensitive cell lines (HL-60 for *KDR *induction, EM-2 for *FLT4 *induction), demethylation did not induce mRNA expression that would translate into protein levels detectable by Western blot analysis (data not shown). These results suggest that other mechanisms than DNA methylation are also involved in the regulation of *KDR *and *FLT4*.

**Table 3:** Effect of 5-Aza-dC on expression of *KDR *and *FLT4*

	*KDR*		*FLT4*	
	mRNA	induced by Aza	mRNA	induced by Aza
CMK	pos	-	pos	-
DOHH-2	neg	+	neg	++
EM-2	neg	(+)	neg	+++
HL-60	neg	+++	pos	-
L-82	neg	-	neg	+
SC-1	neg	++	neg	+

Induction of *KDR *and *FLT4 *by 5-Aza-dC (5 μM, 3 d) when compared to untreated control cells: - < 2.5-fold; (+) > 2.5-fold; + > 10-fold; ++ > 40-fold; +++ > 160-fold. Expression levels were determined by qRT-PCR. *TBP *expression was used as endogenous control, DMSO (0.05%) treated cells were used for normalization

Besides DNA methylation, also histone modifications are epigenetic mechanisms that affect the expression of individual genes. Just to mention two examples, acetylated histone H3 (at lysine 9 and 14) is a marker for gene activation [23], tri-methylation of histone H3 lysine 27 stands for gene suppression [24]. Furthermore, epigenetic modifications can influence each other: methylated CpGs in a promoter region can be targeted by proteins that interact with histone deacetylases. The consequence is an inactive chromatin status and transcriptional repression [25,26].

However, besides epigenetic mechanisms, also the presence or absence of trans-acting factors may govern the expression of *KDR *and *FLT4*. Thus, it has been shown that transcription factor binding sites (Sp1, AP-2 and NFκB) are essential for the base-line activity of the *KDR *promoter [27]. Here, we set out to find whether NFκB also plays a role for the expression of *FLT4*.

### Influence of transactivating factors

During inflammation, new lymphatic vessels are formed. NF-κB is a key mediator of inflammatory processes and has recently been identified as inducer of *FLT4 *on lymphatic endothelial cells [13]. To test whether NF-κB contributes to *FLT4 *expression in leukemic cells, we stimulated the *FLT4 *negative cell line EM-2 and the *FLT4 *positive cell line OCI-AML1 with synthetic MALP-2. MALP-2 binds to toll-like receptors-2 and -6 [28]. MALP-2 triggers the NF-κB pathway [29] which leads to the expression of NF-κB targets like *TNFα *[30].

**Figure 6:**
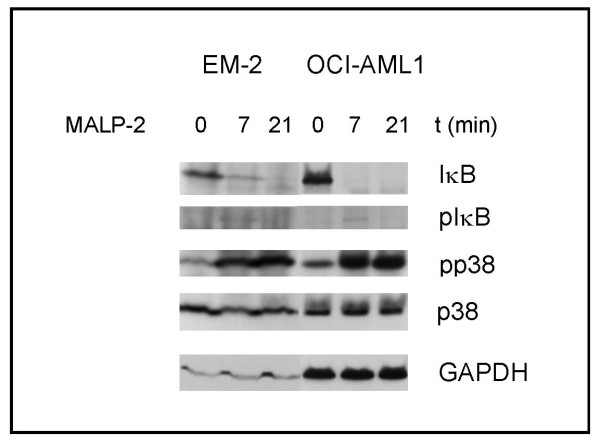
**Degradation of IκB and phosphorylation of p38MAPK in MALP-2 responsive cell lines**. Cell lines EM-2 (FLT4 negative) and OCI-AML1 (FLT4 positive) were stimulated with MALP-2 (100 ng/ml). The NF-κB inhibitor IκB was phosphorylated (7 min) and degraded (7 min, 21 min) in both cell lines. Likewise, rapid phosphorylation of p38 MAPK was observed in both cell lines by Western blot analysis. GAPDH is shown as loading control.

Accordingly, MALP-2 (100 ng/ml, 7 min) induced phosphorylation and degradation of the NF-κB inhibitor IκB and stimulated phosphorylation of p38 in cell lines EM-2 and OCI-AML1 (Figure 6). MALP-2 (100 ng/ml, 1 h) triggered expression of the NK-κB targets *TNFα *(80× in EM-2, 1000× in OCI-AML1) and *IP-10 *(600× in EM-2, > 1000× in OCI-AML1) in both cell lines. However, the expression of *FLT4 *was not affected, neither in the *FLT4 *positive cell line OCI-AML1 nor in the *FLT4 *negative (methylated) cell line EM-2. MALP-2 did also not increase the *FLT4 *stimulating effect of 5-Aza-dC on EM-2 cells (data not shown). Thus, our results do not support the view that NF-κB is a transactivator of *FLT4*. We observed a 10-fold increase in *KDR *in cell lines OCI-AML1 and EM-2 (the latter pretreated with 5-Aza-dC). However, as the level reached after stimulation was still extremely low, it appears unlikey that NF-κB is an important regulator for *KDR *either.

### KDR and FLT4 in HDMECs and HUVECs

**Figure 7:**
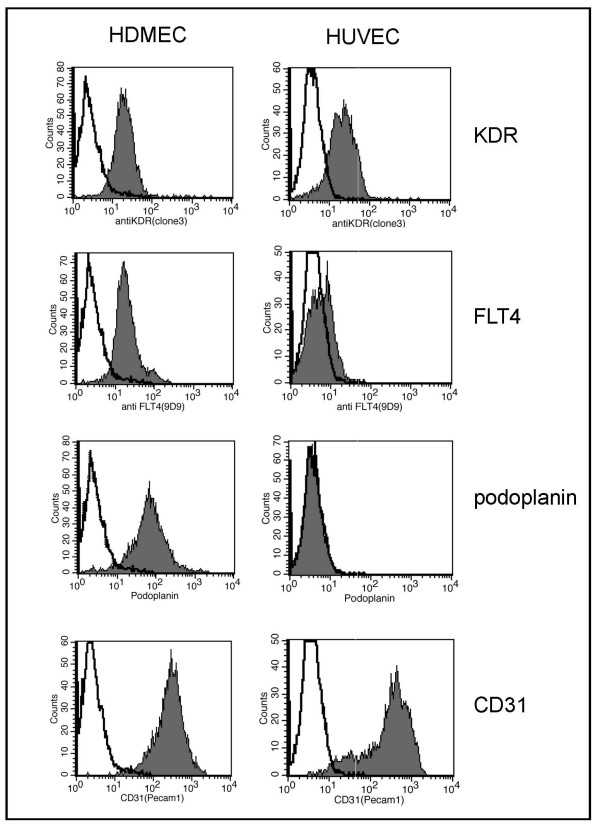
**Expression of KDR and FLT4 on HDMECs and HUVECs**. Flow cytometry analysis for KDR, FLT4, the lymphatic vessel marker podoplanin and the panendothelial marker CD31. Note that HDMECs and HUVECs show comparable KDR expression levels, while FLT4 is stronger in lymphatic vessel cells (HDMECs) than in blood vessel cells (HUCECs). The non-filled peak shows the corresponding isotype control.

HDMECs, HUVECs and *KDR *positive leukemia cell lines exhibited demethylated *KDR *promoters (Figure 3, Table 2). However, mRNA and protein levels were distinctly higher in the primary cells than in the leukemia cell lines (Figure 1). Results of Western blot analysis were confirmed by flow cytometry (Figure 7). HDMECs and HUVECs expressed the pan-endothelial marker CD31 (Figure 7). HDMECs, primarily consisting of lymphatic endothelial cells, were also positive for the lymphatic vessel marker podoplanin, HUVECs were podoplanin negative (Figure 7). Both types of primary cells expressed much higher levels of KDR than KDR-positive cell lines (Figure 1). Also FLT4 expression levels varied greatly from one cell type to the other: FLT4 expression of HUVECs was comparable to those of FLT4 positive cell lines, while HDMECs showed much higher FLT4 expression levels (Figures 1 and 7). These results are in line with our data of MSP analyses and 5-Aza-dC experiments suggesting that DNA methylation is not the only mechanism that controls *KDR *and *FLT4 *gene expression.

## Conclusions

Our data obtained from primary endothelial cells and from leukemia/lymphoma cell lines show that *KDR *and *FLT4 *are epigenetically regulated genes. Both genes can be silenced by methylation. However, if the promoters are unmethylated, other factors are responsible for the extent of KDR and FLT4 expression. Furthermore, we show that the KDR negative/FLT4 positive cell line OCI-AML1 is a model system for FLT4 signal transduction studies.

## Competing interests

The authors declare that they have no competing interests.

## Authors' contributions

HQ designed the study, performed data analyses and wrote the manuscript. SE carried out bisulfite conversion and helped to design primers for M-PCR and U-PCR. JR performed Western blot analyses. HAW supplied antibodies, HDMECs and HUVECs and gave good advice. MZ performed PCR and FACS analyses. HGD provided cell lines and good advice. All authors read and approved the final manuscript.

## Pre-publication history

The pre-publication history for this paper can be accessed here:

http://www.biomedcentral.com/1471-2407/12/19/prepub
